# The ethics of DNR-decisions in oncology and hematology care: a qualitative study

**DOI:** 10.1186/s12910-020-00508-z

**Published:** 2020-07-31

**Authors:** Mona Pettersson, Mariann Hedström, Anna T. Höglund

**Affiliations:** Department of Public Health and Caring Sciences, Uppsala University, Box 564, 751 22 Uppsala, Sweden

**Keywords:** Allow for natural death, Do not resuscitate, Ethics, Oncology, Hematology, Nurses, Physicians, Sweden

## Abstract

**Background:**

In cancer care, do not resuscitate (DNR) orders are common in the terminal phase of the illness, which implies that the responsible physician in advance decides that in case of a cardiac arrest neither basic nor advanced Coronary Pulmonary Rescue should be performed. Swedish regulations prescribe that DNR decisions should be made by the responsible physician, preferably in co-operation with members of the team. If possible, the patient should consent, and significant others should be informed of the decision. Previous studies have shown that physicians and nurses can experience ethical dilemmas in relation to DNR decisions, but knowledge about what ethical reasoning they perform is lacking. Therefore, the aim was to describe and explore what ethical reasoning physicians and nurses apply in relation to DNR-decisions in oncology and hematology care.

**Methods:**

A qualitative, descriptive and explorative design was used, based on 287 free-text comments in a study-specific questionnaire, answered by 216 physicians and nurses working in 16 oncology and hematology wards in Sweden. Comments were given by 89 participants.

**Results:**

The participants applied a situation-based ethical reasoning in relation to DNR-decisions. The reasons given for this were both deontological and utilitarian in kind. Also, expressions of care ethics were found in the material. Universal rules or guidelines were seen as problematic. Concerning the importance of the subject, nurses to a higher extent underlined the importance of discussing DNR-situations, while physicians described DNR-decisions as over-investigated and not such a big issue in their daily work.

**Conclusion:**

The study revealed that DNR-decisions in oncology and hematology care gave rise to ethical considerations. Important ethical values described by the participants were to avoid doing harm and to secure a peaceful and “natural” death with dignity for their dying patients. A preference for the expression “allow for natural death” instead of the traditional term “do not resuscitate” was found in the material.

## Background

A Do Not Resuscitate (DNR) order implies that the responsible physician in advance decides that in case of a cardiac arrest neither basic nor advanced Coronary Pulmonary Rescue (CPR) should be performed to a patient. The reason is that CPR in case of a cardiac arrest is not considered justified, as it would not provide the patient with increased quality of life, but rather increase harm and suffering. Further, the patient can have expressed a desire for such a decision earlier in his/her care. Hence, a DNR order may imply that the patient does not want to be resuscitated, even if the physician’s judgements is that CPR can be performed and is justified.

Swedish regulations prescribe that DNR decisions should be made by the responsible physician [1]. If possible, the patient should consent, and significant others should be informed of the decision. Further, the decision should be made in co-operation with other members of the team [2]. Exactly how this co-operation should be performed is not described in the guidelines. Nurses can initiate and take part in a discussion on the need for a DNR decision, but the responsibility for making the decision lies on the physician. According to the Swedish Council of CPR, CPR must start within 60 s if no DNR decision is in place [3, 4]. Therefore, it is crucial that a DNR-decision is thoroughly documented, so that the team members know whether they should start CPR or not.

Studies have shown that the rules and guidelines for DNR-decisions in Sweden are not always followed [5–7]. International studies have reported that patients prefer to take active part in their DNR-decision [8, 9]. Further, it has been reported that even though patients had expressed that they preferred a DNR-decision in their end-of-life care, in many cases no decision was taken before their death [10]. Thereby there is a risk that two important ethical goals for DNR-decisions are not fulfilled; namely to avoid unnecessary suffering and to respect patient autonomy [11].

Previous research has also investigated the decision-making process around DNR-orders, focusing upon problems experienced by nurses concerning, e.g., when the discussion should be initiated and by whom, who should take part in the decision-making and who should be informed about the decision [10, 12, 13].

As DNR-decisions are quite common in cancer care, research has also addressed this area [8, 14]. In a previous study, we found that several aspects related to DNR-decisions could prevent nurses within oncology and hematology from performing good nursing care [15], such as unclear documentation, differing views in the team, and whether patients and significant others were informed or not of the decision. Further, several examples of ethical dilemmas in relation to DNR-decisions in cancer care were found, such as conflicts of interest between the wish to do good and reduce suffering in the patient versus prolonging life and respect patient autonomy [15]. Hence, ethics tend to be a central part of DNR-decisions, as dilemmas occur frequently in these situations.

Apart from finding that ethical aspects are relevant in DNR decisions, a previous study revealed that a special kind of ethical competence is required in relation to DNR-decisions in cancer care, for both nurses and physicians. The competence found consisted of aspects such as knowledge, virtues and experience, as well as a conscious approach to ethical guidelines [16].

An ethical dilemma can arise from conflicting values, norms and interests, where there may be good reasons for more than one course of action. A choice has to be made, and the loss of at least one value or interest is unavoidable. Therefore, ethical decisions need to be based on good reasons. Traditionally, actions are guided as right or wrong according to consequences or rules. In *utilitarian theories* consequences are at the fore and in *deontological theories* actions are judged according to their conforming to ethical rules or duties [17].

Utilitarianism and deontology are reflected in the well-established ethical principles of *autonomy, non-maleficence, beneficence* and *justice* [18]. From deontological reasoning, the principles of autonomy and justice are derived. In short, these principles prescribe that the moral agent has a duty to respect human dignity and to treat everybody as equals, regardless of consequences of the performed actions. From utilitarian reasoning, the principles of non-maleficence and beneficence have been developed. They learn that the preferred action is the one that increases the total wellbeing of all concerned parties. Maximizing good consequences and limiting harm is the goal.

The hitherto described theories focus on how to act in ethical dilemmas. Another ethical tradition focus on *the character* of the moral agent; namely *virtue ethics*. The argument behind this theory is that virtous individuals are inclined to act morally. Virtues are defined as desirable character traits and they are supposed to be learned through role-models and good examples. Important virtues are, for example, patience, courage, self-reflection and empathy [19, 20].

Another way of structuring ethical theories is according to rules or situations. Situational ethics (or particularism) is characterized by the view that ethical decision-making should be based upon the circumstances of a particular situation [21], whereas rule ethics holds that a person’s conduct should be guided by “universal laws”, i.e., moral laws that hold without exception, in all circumstances [17], (p. 120). Deontological and utilitarian arguing can be of both kinds: situation-based or rule-based [22].

One form of ethics that has become influential since the 1980s is the *ethics of care* [23, 24]. The experience of giving and receiving care is the starting-point for this tradition, and care is regarded as a moral value, as well as a practice. The idea within this tradition is that we should strive to meet the needs of “particular others”, for whom we are responsible [25]. In care ethics, individuals’ interdependence is acknowledged. The view of the moral agent thereby differs from the one in utilitarianism and deontology, where people are supposed to be independent, rational and autonomous. Care ethics, on the contrary, learn that we are all embedded in social contexts, characterized by power orders related to factors such as socio-economy and gender.

The described ethical approaches are reasonably applicable on DNR-decisions in cancer care, although few studies have investigated which of these are actually applied in such situations. More knowledge is needed on what kind of ethically troubling situations DNR decisions include, as well as on how physicians and nurses in cancer care reason around such dilemmas. Therefore, the aim of the present study was to describe and explore what ethical reasoning physicians and nurses in oncology and hematology care apply in relation to DNR-decisions.

## Method

### Design

A qualitative, descriptive and explorative design was used, based on free-text comments in a study-specific questionnaire, provided to physicians and nurses working in oncology and hematology wards in Sweden.

### Material

The present study is part of a larger project, investigating clinical and ethical perspectives of DNR-decisions in oncology and hematology care. Within the project, a study-specific questionnaire in the form of a web-survey was developed, to measure nurses’ and physicians’ views on the process of DNR-decisions within oncology and hematology care and what values they found important for well-grounded DNR-decisions. The questionnaire started with a short vignette, adapted to either oncology or hematology. It described a cognitively clear but terminally ill 75-year male, for whom a DNR discussion was pertinent. Thereafter, the respondents answered questions on who should participate in DNR decisions, to whom the decision should be informed, and how the decision should be documented [7]. For each topic, the respondent first estimated (on a 6-step Likert scale) how *important* a part of the DNR process was and in the next part estimated how *likely* this part was to happen at the ward. The respondents also chose values most important when deciding on DNR [7]. The detailed procedure and the results from the questionnaire are presented elsewhere [7].

At the end of each topic in the questionnaire, the participants had the opportunity to write free text comments in relation to the current topic and at the very end of the questionnaire they were allowed to give free comments, independent of topic. The present article describes the results from the analysis of all free text comments.

### Participants

The study was performed at seven hospitals in mid-Sweden. Sixteen hematology and oncology departments were included in the study. The study specific web-survey was sent to 295 nurses and 206 physicians. The response rate was 45% (*n* = 132) for nurses and 41% (*n* = 84) for physicians. Hence, a total of 216 participants answered the questionnaire [7]. A total of 287 comments were given by 89 participants (41.2%) in the study. They had a mean age of 42 years (range 22–67 years). There were 46 nurses (15 within hematology, and 31 within oncology), and 43 physicians (14 within hematology and 29 within oncology). No significant differences were found between those who wrote comments and those who did not regarding age, gender or working years. However, comments were more frequent among physicians compared to nurses (χ^2^ = 5.6; *p* ≥ 0.05). Characteristics of the participants are presented in Table 1.

**Table 1:** Characteristics of participants

		***n***	Total	Working in hematology	Working in oncology
**Nurses**	n (%)	*46*	46	15 (33%)	31 (67%)
Age	M (range)	*45*	38 (22–66)	38 (26–64) *n = 14*	39 (22–66) *n = 31*
Gender	F/M (%)	*45*	43/2 (96/4%)	13/1 (93/7%)	30/1 (97/3%)
Years in profession	M (range)	*36*	11 (0.5–44)	11 (0.5–32) *n = 14*	11 (0.5–44) *n = 31*
Specialist training	Yes (%)	*N/A*	9 (20%)	4 (27%)	5 (16%)
Years in oncology/hematology	M (range)	*36*	N/A	7 (1–19) *n = 11*	8 (0–27) *n = 25*
**Physicians**	n (%)	*43*	43	14 (33%)	29 (67%)
Age	M (range)	*43*	45 (27–67)	47 (30–67)	44 (27–65)
Gender	F/M (%)	*43*	24/19 (56/44%)	5/9 (36/64%)	19/10 (66/34%)
Years in profession	M (range)	*43*	17 (1–41)	20 (8–41)	16 (1–38)
Specialist training	Yes (%)	*N/A*	37 (86%)	14 (100%)	23 (80%)
Years in oncology/hematology	M (range)	*43*	N/A	12 (1.5–30)	11 (0.5–33) *n = 28*

### Procedure

Data was collected from February to October, 2017. Ward managers or other coordinators provided e-mail addresses to nurses and physicians who had worked in oncology and/or hematology wards for at least 6 months. Then information about the study, including a link to the web-survey was sent. At least two reminders were sent to all, and several wards were also provided with paper surveys, as reported in previous published work [7].

### Analysis

The free text comments were gathered verbatim in a separate document. The comments were analyzed using inductive thematic content analysis [26]. All comments were read through thoroughly and meaning units answering the aim of the study were identified and grouped together and sorted into theme, categories and sub-categories. The categories and subcategories can be described as the manifest content of the data. In line with Graneheim and Lundman [27], we consider a theme to be “a thread of an underlying meaning through condensed meaning units, codes or categories, on an interpretative level” [27] (p. 107). This means, that the theme can be seen as an expression of the latent content of the data.

### Ethical considerations

Approval for the study was sought at the Regional Ethics Review Board (Dnr 2016/484). However, with reference to the Swedish Act on the Ethical Review of Research Involving Humans [28] the Board found that no formal approval of the project was needed as the study did not deal with sensitive personal data or risked impact the participants, physically or psychologically.

National and international guidelines and regulations for empirical research were followed [28, 29]. Permission to perform the study was given by the Head of Department of the respective clinics in each hospital. Physicians and nurses received information on the study and by responding to the survey, they consented to participate [7].

## Results

In the 216 distributed questionnaires, 287 comments were made. The number of comments for each questionnaire topic is described in Table 2.

**Table 2:** Number of comments on each questionnaire topic

Topic	Number of comments (***n*** = 287)
Consultation with patient	50
Consultation with relatives	36
Consultation with other physicians	40
Consultation with nurses	31
Information to patient	33
Information to significant others	21
Information to team	31
Documentation	18
Important values	8
Other comments	19

**Fig. 1:**
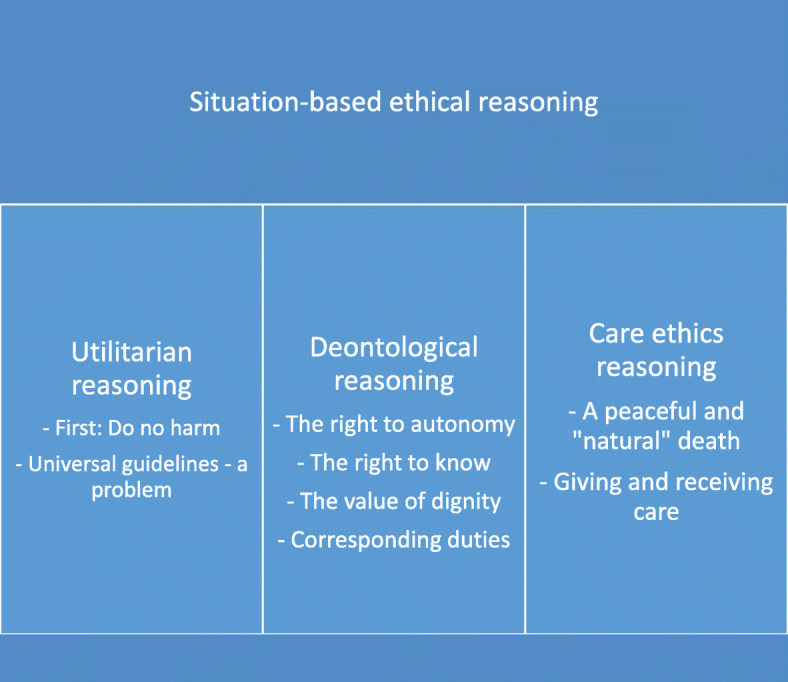
Overview of theme, categories and sub-categories derived from the content analysis

Through the analysis, one overarching theme and three categories with subcategories were developed. An overview of theme, categories and subcategories is presented in Fig. 1.

In the following, theme and categories will be described and illustrated by quotes.

### Theme: situation-based ethical reasoning

As an overarching theme, situation-based ethical reasoning was derived. The participants described how DNR-decisions were complicated issues, why they strived to adopt to the specific situation. Primarily, questions of information to patient and/or significant others, as well as consent and shared decision-making in relation to DNR, were apprehended as difficult matters and therefore the ethical decisions had to be based upon circumstances in every particular situation. Hence, the participants described how they advocated a situation-based ethics in relation to DNR-decisions.

Difficult questions but, of course, important! But much depends on the situation. (Nurse 182, oncology)

It all depends on the patient. (Physician 26, oncology)

The participants expressed varying opinions on the importance of DNR-decisions. Some held that DNR is an important topic to discuss and research, while others regarded it as a question that had received far too much attention.

An interesting and well-documented topic, but hardly a problem in our every-day work. (Physician 114, oncology)

DNR-decisions is a sensitive issue in the media and in politics, but in reality, it’s not so difficult. To go from curative to palliative care, on the other hand … . can be a tricky decision, both medically and ethically. (Physician 160, hematology)

Extremely important topic to research, discuss and debate! (Nurse 167, hematology)

Concerning consultation with members of the team around the patient (nurses and physicians) the respondents described how this varied according to the responsible physician’s experience and competence. Experienced physicians did not need to consult with others, according to the participants.

It depends on the physician’s competence. (Physician 1, oncology)

A physician who is experienced in making DNR decisions does not need to consult with others. But, if s/he is not used to making these decisions, it’s always best to consult several colleagues. (Nurse 16, oncology)

Further, the severity of the decision could also impact whether consultation with colleagues was sought or not.

Sometimes, these decisions are easy, but in some cases, they are difficult and then it’s best to discuss it with colleagues. (Physician 139, hematology)

Also, whether the team was involved in the decision or not varied, depending on the routines at the ward at stake.

I have experienced that the physicians asked for my opinion and I really appreciate that. But, it could be done more frequently! (Nurse164, hematology)

If the nurse knows the patient better, the nurse’s input can be valuable. (Physician 132, oncology)

### Category 1: utilitarian reasoning

The situational ethics that was described by the participants was expressed in different ways. One kind of reasoning in order to justify the situation-based decisions was of utilitarian type. Two subcategories were developed within this category: the ambition to avoid doing harm to the patients and a doubtful attitude towards universal rules and guidelines.

#### First: do no harm

From an ethical point of view, it is important to distinguish between the decision that CPR is not desirable and the decision that discussing CPR with a patient is not desirable. The respondents declared that, even though shared decision-making and informed consent concerning DNR might be desirable, ethical reasons made it sometimes inappropriate. Primarily, a wish to avoid suffering or doing harm, in the form of causing stress or fear in the patient, were given as reasons for such a situation-based approach.

If the patient wants to discuss CPR we should do it. Otherwise, it can be cruel and inhumane to bring up this question. (Physician 79, oncology)

It might cause unnecessary stress for both patient and relatives if they somehow get the impression that they are responsible for a DNR-decision. (Physician 84, oncology)

The most important thing is to not harm the patient. And it can be to harm the patient if a DNR-decision does not consider expected chance of survival and quality of life after CPR. (Nurse 180, oncology)

#### Universal guidelines - a problem

Another expression of the situation-based ethical reasoning in this category was the participants’ approach to national and local guidelines for CPR and DNR in Sweden. They expressed how such guidelines were not always helpful, due to their general character. The participants seemed to lack more contextualized guidelines for their own practice.

The guidelines for DNR are made for other specialties, where it’s not as obvious that the patient is dying from his/her disease. (Physician 147, oncology)

### Category 2: deontological reasoning

In contrast to the above category, the free-text comments also included expressions of the need for principles and duties of a more universal kind around DNR-decisions, i.e., expressions of a deontological form of ethical reasoning. Within this category, four sub-categories were found, including the patient’s right to autonomy, information and dignity, as well as duties related to these rights.

#### The right to autonomy

The participants described certain rights that the patients were entitled to. Primarily, the right to autonomy was at the core of these expressions.

The patient’s self-determination is extremely relevant, if s/he expresses a wish for a DNR-decision. (Physician 82, oncology)

#### The right to know

The participants also described how they regarded it a central right for the patient to get information and thereby get the possibility to consent or not to a DNR-decision.

The patient is entitled to information on DNR. S/he has the right to a conversation with the physician, including information on his/her medical prognosis and an explanation of what DNR is and what it would mean for his/her condition. (Nurse 83, hematology)

I wish patients were informed on every DNR-decision, but my experience is that this is not always the case. (Nurse 154, oncology)

It’s always important for relatives to get information about the decision and on what grounds it has been made. (Nurse 16, oncology)

#### The value of dignity

As described above, autonomy was put forward as an important right of the patient. It was also sometimes described as a value among the participants. Apart from autonomy, dignity was a value that was emphasized by the informants.

I have seen patients being treated until the very end, and that is not always dignified. I think that every person has the right to a dignified end of life. (Nurse 189, oncology)

Severely ill patients must have the possibility to end their lives in a dignified way. They should not have to die connected to ventilators in an intensive care unit after a cardiac arrest. (Physician 147, oncology)

#### Corresponding duties

When there are rights, there are duties, and the participants expressed that it was the physician’s duty to ensure the patient’s right to autonomy and informed consent.

As I see it, it is the physician’s duty to inform the patient and/or significant others in a way that makes them understand what a DNR-decision would mean for the patient. /--/ I think it is the physician’s task to consider the ethics of the decision … (Nurse 83, hematology)

DNR is about tidiness and regularity. It’s about security for the nurse and safety in care and dignity for the dying patient. (Nurse 123, hematology)

A prerequisite for such tidiness was a correct documentation of the decision and that everyone in the team was aware of the decision.

There mustn’t be uncertainty on what is decided. (Physician 79, oncology)

The decision must be clearly documented, so that all staff is informed. (Physician 131, oncology)

### Category 3: care ethics reasoning

Finally, the analysis revealed expressions of care ethics, in that the participants described how their ethical judgements were based on a will to provide good care to the dying patient. The patients were regarded as vulnerable, as they were in the end of their lives, and thereby the ethical demand first and foremost seemed to be to perform good care for the dying patient. Two sub-categories were developed in relation to this category, namely the participants’ striving for providing their dying patients a peaceful and natural death and their experience of giving and receiving care.

#### A peaceful and “natural” death

Not least, the wish for a peaceful death for terminally ill and dying patients was mentioned as an important ethical aspect within this category.

Severely ill patients should rather pass away peacefully, adequately sedated and with significant others at their side. (Physician 147, oncology)

I know a case when a very young patient had a DNR-decision on the ward, but was given CPR in the ambulance and in the emergency room. /---/ Also, a very old woman without a DNR-order who was resuscitated by the staff. She ended up with care for broken ribs for a month and she was very upset that she was not allowed a peaceful death. (Nurse 151, hematology)

In the material, the expression “a natural dying process” was used when the participants referred to aspects of a peaceful death.

If the patient is terminally ill, I consider CPR as unmotivated or unethical; a medical intervention that risks prolonging the natural dying process and thereby not benefiting the patient. (Nurse 83, hematology)

Further, the expression “a natural death”, instead of a peaceful death, was used in the material, and the participants described how they would prefer that expression to DNR.

Most of our patients are so ill that their chances to survive a cardiac arrest are very small, even with immediate CPR. /---/ Therefore, I suggest that the documentation of the decision should be “allow natural death” instead of DNR. (Physician 25, oncology)

#### Giving and receiving care

Another expression of care ethics was the described will to provide considerate care to terminally ill patients. An expression of that were quotes that described how, instead of taking up a discussion on DNR, the participants preferred to give information on what would actually be done for the dying patient, instead of what was going to be refrained from.

… we need to create a calm and secure atmosphere around the patient. (Physician 131, oncology)

It’s important to inform patients and significant others that we do everything we can until the cardiac arrest, for example pain relief, antibiotics etc. (Nurse 53, oncology)

## Discussion

The aim of the present study was to describe and explore what ethical reasoning physicians and nurses apply in relation to DNR-decisions in oncology and hematology care. The results revealed that when it came to DNR-decisions the participants primarily applied some kind of situation-based ethical reasoning. The arguments given in various dilemmas were both deontological and utilitarian in character. The strive to first of all avoid doing harm to the dying patient can be seen as a utilitarian arguing, while the referring to the patient’s right to autonomy primarily is of deontological character [17].

The reasons given by the participants for such a situation-based ethics was first and foremost that universal rules or guidelines could be problematic. This has also been shown in previous studies [30, 31]. Also, in a previous study we found that rules and guidelines on DNR- orders are only useful if those who should use them already possess a certain ethical competence [16]. However, the participants did adhere to some values and rights as universal, namely the rights to autonomy, information and dignity. Primarily, the patient’s right to a peaceful death, with dignity, was put forward by the participants. A peaceful death has been highlighted by, among others, Virginia Henderson in her theory of nursing [32]. A previous study further found that nurses adhered to this view when participating in DNR-situations in cancer care [15].

Examples of care ethics were also found in the material, expressed by both physicians and nurses. One could discuss whether these examples are best described as an ethics of care or as a kind of virtue ethics. According to Michael Slote [33], the ethics of care is a form of virtue ethics. Against this, however, one can argue that the ethics of care differs from virtue ethics in that it is less focused on individuals and their developing of character, and more interested in relations and interdependence between people. This has been put forward by, for example, Virginia Held [25].

Our results showed that the participants strived to provide considerate care to their terminally ill patients. One example of this was comments that described how they used to emphasize what they would do to provide a calm and peaceful death for the patient. That could sometimes lead to a refraining from taking up a discussion on DNR. This can be interpreted as an ethical reasoning where the the focus is on caring for others as well as on relations and interdependence [25].

A critique that has been provided against the ethics of care ever since the 1990s is that it might be paternalistic, in that you perform what you think is good and caring actions towards someone else, without assuring that they apprehend it as such [34]. This has led to a development within the ethics of care. Among advocates of care ethics, it is now often underlined that a moral action, in order to be interpreted as an act of care, must be apprehended as such by the recipient. In other words, it is only care if it is experienced as care [35].

At first glance, the ethics of care, which emphasizes the importance of avoiding that anyone gets hurt, might seem as the same thing as the principle of non-maleficence or the principle of beneficence, as described by Beauchamp and Childress [18]. However, the ethics of care, with its focus on relations, differs from principled-based ethics’ “generalized beneficence”, as Anne Donchin [36] puts it. Her point is, that the principle of beneficence can be interpreted as “disinterested care” directed to “undifferentiated others”. Against this, the ethics of care is based on a certain view of the moral agent, namely as a person who is embedded, dependent and relationally constructed [25].

In line with the ethics of care, the results in this study can be interpreted as a doubtfulness towards a principle-based ethics among the participants, such as the ethics developed by Beauchamp and Childress [18]. Rather than referring to universal rules that hold without exception in all circumstances [17] (p. 120) the participants expressed a context sensitivity that resulted in a situation-based ethics approach.

Concerning the importance of discussing decisions on DNR, different opinions were found in the material. The different positions were slightly connected to profession, in that nurses to a higher extent than physicians underlined the importance of the subject, while physicians expressed that DNR had been almost over-investigated in the last few years and that it was not such a big issue in their daily work. In light of the Swedish guidelines, this is reasonable, as the nurses must perform CPR within 60 s in case of a cardiac arrest, if no DNR decision is in place. That nurses therefore need clear and well-documented decisions concerning DNR has previously been reported [15].

Finally, a preference for the expression “allow for natural death” (AND) instead of “do not resuscitate” (DNR) was found in the material. The term AND has been proposed as a “softer” and “warmer” term than DNR, easier to accept for patient and families [37, 38]. The reason behind this new terminology was to ensure patients to die with dignity, i.e., a highly ethical motive [38]. Nevertheless, the expression can be discussed. For example, one can ask what “natural” really means. If something is possible to do, can it then, at the same time, be unnatural? And if something is apprehended as “unnatural”, does that mean that it is also morally wrong? And, most of all, one can question whether the terminology really changes how patients are treated.

However, in line with the results in the present study a change of terminology, from DNR to AND, could be reasonable, as it might be apprehended as a softer and less sensitive expression. Thereby, it could help the responsible staff to adhere to the ethical guidelines that urge them to inform patient and significant others about the decision.

The clinical implications of the study is that staff involved in DNR decisions in cancer care need to be able to express the ethical reasons behind their decisions. Therefore, education and learning in the context where the decisions are made is necessary. From an ethical point of view, the requirement for both adherence to principles, such as autonomy and informed consent, and the striving for good consequences, such as avoiding harm in the patient, are the most important ones when it comes to DNR decisions in cancer care. Apart from that, an attitude of caring for other persons, seeing them as moral agents, embedded in relationships and in need for compassion and care, is what really matters in end of life care.

## Strengths and limitations

The study was qualitative, based on a very rich material from a questionnaire distributed to sixteen wards of various sizes in Sweden. Data included 287 free text comments that formed the basis for the content analysis. We argue that the results are transferrable to similar contexts, i.e., wards where DNR-decisions are frequently made, situated in countries with health care systems similar to the Swedish one.

## Conclusion

DNR-decisions are frequently made in cancer care and our study showed that these decisions require serious ethical consideration. The participants expressed a situation-based ethical reasoning and justified their decisions with reference to arguments that can be interpreted as both utilitarian and deontological in character. Expressions of care ethics were also found in the material. Some ethical values were highly emphasized by the participants, namely avoiding harm and securing a peaceful and “natural” death with dignity for their dying patients. Further, a preference for the expression “allow for natural death” instead of the traditional term “do not resuscitate” was found in the material.

## Data Availability

The datasets used and/or analyzed in the current study are available from the corresponding author on reasonable request.

## Abbreviations

AND: Allow for Natural Death
CPR: Cardiopulmonary resuscitation
DNR: Do-not-resuscitate
